# A Novel Identified Long Intergenic Noncoding RNA, LINC01574, Contributes to Breast Cancer Deterioration via the Regulation of miR-6745/TTYH3 Axis

**DOI:** 10.1155/2022/4201283

**Published:** 2022-07-27

**Authors:** Liang Zhang, Lingyuan Wu, Mengjiao Wei, Peikai Ding, Xingsong Tian, Kunbing Zhu

**Affiliations:** ^1^School of Medicine, Cheeloo College of Medicine, Shandong University, Jinan, Shandong 250012, China; ^2^Department of Breast and Thyroid Surgery, Shandong Provincial Maternal and Child Health Care Hospital, Jinan, Shandong 250014, China; ^3^Reproductive Medicine Center, Shandong Provincial Maternal and Child Health Care Hospital, Jinan, Shandong 250014, China

## Abstract

**Objective:**

Compelling evidence suggested that lncRNAs performed vital functions in the development of breast cancer (BC). The study intended to mine the functional roles of LINC01574 in BC and further excavated its underlying regulatory mechanism.

**Methods:**

The expression and prognosis of LINC01574 in BC were detected by integrating analysis of data mining, bioinformatics, and RT-qPCR. Then, the effect of LINC01574 knockdown on BC cell growth and metastasis was evaluated in vitro and in vivo. Interactions between miR-6745 and LINC01574 or TTYH3 were revealed by both target prediction and dual luciferase reporter assay.

**Results:**

Our data found that LINC01574 was markedly elevated in BC tissues and cells and was an independent prognostic risk factor for patients with BC. Further functional studies revealed that knockdown of LINC01574 remarkably inhibited the growth and metastasis of BC cells in vitro and in vivo. Mechanistically, LINC01574 competitively binds with miR-6745 to prevent the degradation of TTYH3, thereby promoting the development of BC.

**Conclusion:**

Our results unmasked a novel LINC01574/miR-6745/TTYH3 regulatory axis in BC progression and suggested that LINC01574 might be a promising prognostic indicator and therapeutic target for patients with BC.

## 1. Introduction

Breast cancer (BC) is a known refractory tumor with a high coverage rate in women, usually occurring in the glandular epithelium of the breast, accounting for about 31% of all female cancers [1–4]. The factors that induce BC are complex, such as age, environment, physical activity, and genetics, which can trigger the occurrence of BC [5, 6]. Accumulating data indicate that the incidence of BC is gradually increasing in developing countries, including China [7]. Surgery, radiation therapy, chemotherapy, and molecular therapy are currently the more feasible treatment options for patients suffering from BC. However, on account of lacking attention to clinical examination of BC, a considerable degree of patients were already in advanced stages at the time of their visit [8]. Furthermore, not all patients can benefit from these treatments, and the prognosis is still far from satisfactory [9]. Therefore, identifying more effective BC therapeutic targets and exploring the underlying mechanisms behind BC are of considerable significance for precise targeted therapy.

Long noncoding RNAs (lncRNAs) are a kind of regulatory molecules, which play biological functions in the form of RNA [10]. Increasing evidence has indicated that lncRNAs dysregulation is associated with a variety of diseases, and its association with these diseases can contribute to providing insights into disease pathogenesis, diagnosis, and treatment [11]. Besides, lncRNAs have been shown to adjust genes and participate in tumor progression at multiple levels, including BC [12]. For example, Lu et al. demonstrated that lncARAP1-AS1 could accelerate BC process through affecting the miR-2110/HDAC2/PLIN1 axis [13]. Mao et al. proved that LINCRNA 00641 can hold up the BC malignant phenotype through sponging miR-194-5p [14]. As a novel lncRNA, LINC01574 was proved to be associated with glioblastoma [15]. However, the function and underlying mechanisms of LINC01574 in BC remain unknown.

Mechanistically, current research shows that lncRNAs regulate gene expression through various mechanisms including chromatin modification, transcriptional control, and posttranscriptional processing, thereby affecting the development of breast cancer [16]. Among them, the most common mechanism is that lncRNAs decrease the suppression of their downstream target gene expression by acting as the competing endogenous RNAs (ceRNAs) to sponge miRNA [17]. MicroRNAs are RNA molecules of about 21 to 23 nucleotides that are widespread in eukaryotes. They come from some RNAs that are transcribed from DNA but cannot be further translated into proteins [18]. miRNAs usually exhibit abnormal expression under different physiological and pathological conditions. Many studies have identified the functions and mechanisms of miRNAs in different diseases, which have been focused and screened as specific biomarkers for disease predictions, including BC [19, 20]. For examples, Majed and Mustafa confirmed that the miR-1275 expression decreased in BC [21]. Zhao et al. suggested that miRNA-205 promoted BC occurrence and was a promising biomarker for the treatment of apoptosis-resistant tumor [22]. In addition, both miRNA-27a and miRNA-138-5p were also identified as *potential diagnostic* markers and therapeutic target of BC [23, 24]. miR-6745 was a newly discovered miRNA that has been reported in metabolic reactions [25]. Notably, a new evidence depicted that miR-6745 participated in gastric cancer as a tumor suppressor [26]. However, its role and mechanism in BC have not been explored.

Hence, we performed the integrated analysis of data mining, bioinformatics, and RT-qPCR and manifested that the high expression of LINC01574 in BC is an independent prognostic risk factor for patients with BC. Further, our data demonstrated that LINC01574 knockdown inhibited BC growth and lung metastasis by regulating the miR-6745/TTYH3 axis. Overall, this study illuminated the function and underlying mechanism of LINC01574 in BC and provided promising prognostic indicators as well as potential therapeutic targets for patients with BC.

## 2. Materials and Methods

### 2.1. Bioinformatics

The differential expression of lncRNAs in BC patients was obtained from The Cancer Genome Atlas (TCGA) and screened by the criteria of ∣logFC | >2 and *P* < 0.05. The clinical information of BC patients from TCGA is presented in Table S1. miRNAs with potential binding sites for LINC01574 were predicted via miRBD, and target genes of these miRNAs were predicted through miRWalk, TargetScan7, and miRDB. The mRNA and protein of TTYH3 in BC were analyzed by TCGA and Clinical Proteomic Tumor Analysis Consortium (CPTAC) databases.

### 2.2. Sample Collection

BC tumor tissues and paired adjacent normal tissues were collected from patients with BC (*n* = 30). Meanwhile, clinical characteristics of the patients (such as age, clinical stage, tumor size, metastasis, and other clinically meaningful indicators) were also collected. Each subject was aware of and has signed written informed consent. This study was approved by Shandong Provincial Maternal and Child Health Care Hospital Ethics Committee (No. 2021-110).

### 2.3. Cell Culture

Normal breast epithelial cell lines (MCF-10A) and BC cell lines (MCF-7, MAD-MB-231, MAD-MB-468, and MDA-MB-453) were purchased from Procell Life Science & Technology Co., Ltd. (Wuhan, China). After the cells were resuscitated, MCF-7 cells were cultured in specific medium (#CM-0149, Procell Life Science & Technology Co., Ltd), and other cells were cultured in L15 medium (#LA9510, Beijing Solarbio Science & Technology Co., Ltd.) for use in subsequent experiments.

### 2.4. Quantitative Real-Time PCR (qRT-PCR)

Total RNA was extracted from tissues and cells using RNA exaction kits (Invitrogen, USA). For mRNAs and lncRNAs, the total RNA was reversed into cDNA by the PrimeScript™ RT Reagent Kit with gDNA Eraser (Takara, Dalian, China); then, qRT-PCR procedure was performed using SYBR Premix Ex Taq™ Kit (Takara) on ABI 7500 RT-PCR system (Carlsbad, USA). GAPDH was used as housekeeping gene. For miRNA expression detection, miRcute miRNA Isolation Kit (Shanghai Zeye Biotechnology Co., Ltd., China) was employed to separate target miRNA, and miRcute Plus miRNA First-Strand cDNA Synthesis Kit (Shanghai Zeye Biotechnology Co., Ltd., China) was used to reverse miRNA into cDNA. On ABI 7500 qRT-PCR system, the expression of miRNA was determined via miRcute Plus miRNA qPCR Detection Kit (Shanghai Zeye Biotechnology Co., Ltd., China). U6 was acted as housekeeping gene for quantification of miRNA. The primer sequences were listed as follows: LINC01574, forward: 5′-TGTCCGCCTTGGGTACAGAAGC-3′, reverse: 5′-GGGATAAACGCCGAATGCCTCT-3′; TTYH3, forward: 5′-AGAACGCTAATTTCCAGAACCC-3′, reverse: 5′-GTGGCGAGGTATTTGGCTCTC-3′; GAPDH, forward: 5′-AAGTATGACAACAGCCTCAAG-3′, reverse: 5′-TCCACGATACCAAAGTTGTC-3′; miR-6745 RT primer, GTCGTATCCAGTGCGTGTCGTGGAGTCGGCAATTGCACTGGATACGACAACCAG; miR-6745, forward: 5′-AAAGCGTGGGTGGAAGAAGGT-3′, reverse: 5′-GTCGTATCCAGTGCGTGTC-3′; U6 primer, AAAATATGGAACGCTTCACGAATTTG; U6, forward: 5′-CTCGCTTCGGCAGCACATATACT-3′, reverse: 5′-ACGCTTCACGAATTTGCGTGTC-3′. The calculation of gene level was realized through 2^-*ΔΔ*Ct^ method.

### 2.5. Subcellular Localization Analysis

The distributions of LINC01574 in BC cells were analyzed by subcellular fractionation assay and fluorescence in situ hybridization (FISH). For subcellular fractionation, total RNA was extracted from the cytoplasm and nucleus using cytoplasmic and nuclear RNA Purification Kit (Shanghai Bangjing Industrial Co., Ltd., China), and then, the LINC01574 expression in the cytoplasm and nucleus was quantitatively determined by qRT-RCR. As a loading control, GAPDH and U6 were for the cytoplasm and nucleus, respectively. For lncRNA FISH analysis, BC cells were first fixed in 4% paraformaldehyde (Henan Tianfu Chemical Co., Ltd., China) and then hybridized with FAM-labeled LINC01574 probe (Geneseed Biotech, Guangzhou, China) overnight at 37°C. After, the cells were stained with 4′, 6-diamidino-2-phenylindole (DAPI) (Tocris Bioscience, UK) and the fluorescence images were then obtained under a fluorescence microscope (Nikon, Japan).

### 2.6. Cell Transfection

The short-hairpin negative control (shNC), shLINC01574 (#1, #2 and #3), NC mimics, miR-6745 mimics, NC inhibitor, and miR-6745 inhibitor were all constructed by RiboBio Corporation (China). The amplified products of TTYH3 were ligated into pc-DNA3.1 to construct overexpression vector (Life Technology, USA). The transfection was realized through Lipofectamine 2000 (Hefei Bomei Biotechnology Co., Ltd., China). Primer sequences were listed as follows: LINC01574 shRNA#1: CCGGGGTCTGCTGTGCATCTTAACTCGAGTTAAGATGCACAGCAGACCTTTTTGAATT; LINC01574 shRNA#2: CCGGTCCCAGACCTAAGTTTGAAGATCCGAAGATCTTCAAACTTAGGTCTGGGTTTTTGAATT; LINC01574 shRNA#3: CCGGTACCATCACAGAGAGCGATAAACTCGAGAATATCGCTCTCTGTGATGGTTTTTTGAATT; sh-NC: CCGGTCCTAAGGTTAAGTCGCCCTCGCTCGAGCGAGGGCGACTTAACCTTAGGTTTTTGAATT; miR-6745 mimics, F: 5′-CCAGACCUUCUUCCACCCAUU-3′; R: 5′-GGGATAAACGCCGAATGCCTCT-3′; NC mimics, F: 5′-UUGUACUACACAAAAGUACUG-3′; R: 5′-GUACUUUUGUGUAGUACAAUU-3′; miR-6745 inhibitor, AACCAGACCUUCUUCCACCCA; NC inhibitor, CAGUACUUUUGUGUAGUACAA.

### 2.7. Mouse Xenograft Model

Nude mouse tumorigenesis model and lung metastasis model were employed to examine the function of LINC01574 on BC cell growth and metastasis in vivo. All animal experiments were approved by the Ethics Committee of Shandong Provincial Maternal and Child Health Care Hospital (No. 2021-111).

For the tumorigenesis model, MDA-MB-231 cells (1 × 10^7^) transfected with shNC or shLINC01574 were first added in 200 *μ*L PBS and then subcutaneously injected into the dorsal and ventral parts of 6-week-old BALB/c nude mice. The model mice were divided into two groups: shNC group (*n* = 5 nude mice) and shLINC01574 group (*n* = 5 nude mice). The weight and tumor volume of nude mice in each group were measured, and the weight curves and tumor growth curves were then drawn. After 4 weeks, the nude mice were euthanized, and the tumor tissues were weighed and partially subjected to H&E staining and immunohistochemistry (Ki-67).

For the nude mouse lung metastasis model, MDA-MB-231 cells (5 × 10^5^) transfected with shNC and shLINC01574 were injected into the same type of mice by the tail vein. Similarly, the grouping of model mice was as described above. Nude mice were weighed every 3 days, and the growth curve of nude mice was then drawn. After 4 weeks, the nude mice were euthanized and then, their lung tissues were harvested and weighed. Meanwhile, the overall structure of lung tissues was observed and the number of tumor was calculated. In addition, H&E staining was used to determine tumor lung metastasis.

### 2.8. Cell Counting Kit-8 (CCK-8)

Cell viability of BC cells was tested by CCK-8 (AbMole, USA). Briefly, the BC cells with the appropriate concentration were inoculated in a 96-well plate. Next, CCK-8 was appended to per well and cultured in the dark for 2 h. The absorbance at 450 nm was detected and represent as cell viability.

### 2.9. Colony Formation Assay

BC cells at the same density were cultured for 14 consecutive days. After washing, the cells were fixed with 4% formaldehyde at 37°C for 40 min. Then, residual formaldehyde was removed and crystal violet (Shanghai Sunwise Chemical Co., Ltd., China) was added for dyeing for 25 min. The colonies were imaged via under an inverted microscope (Carl Zeiss AG, Germany).

### 2.10. Wound Healing Assay

First, BC cells were digested and transferred into 12-well plates. After cell fusion reached 80%, a cell scratch was created through a clean EP tube. Thereafter, serum-free medium was added for cell cultivation. Finally, the changes of the wound surface were observed under an inverted microscope (Carl Zeiss AG, Germany), and pictures were recorded at 24 h.

### 2.11. Transwell Invasive Assay

After serum starvation for 12-24 h, the BC cell suspensions (5 × 10^5^) were prepared. Then, appropriate cell suspension was appended to the upper chamber of a transwell containing 300 serum-free medium coated with Matrigel on the bottom. Meanwhile, 500 *μ*L serum-containing medium was appended into the lower chamber. After conventional culture and fixing, they were stained with 0.1% crystal violet (Henan Tianfu Chemical Co., Ltd., China) for 30 min, respectively. Finally, the number of invasive tumor cells was observed and calculated under an inverted microscope (Carl Zeiss AG, Germany).

### 2.12. Histological Analysis

Histological analysis was performed via hematoxylin-eosin (H&E) staining and immunohistochemistry. For H&E staining, the tissues were first fixed with formaldehyde (10%) for 24 h and then were successively rinsed with water, dehydrated with alcohol, made transparent with xylene, and embedded in paraffin. After that, the slices were stained with hematoxylin (Chengdu GLP biotechnology Co Ltd., China) for 5 min and eosin (Henan DaKen Chemical CO., LTD., China) for 1 min. Finally, the pathological changes of BC tumor tissues and lung tissues were observed under a microscope. For immunohistochemistry, it was conducted using anti-Ki-67 (ab15580, 1 : 750, Abcam) according to the previous protocol [27]. The stained samples were analyzed using an Olympus microscope.

### 2.13. Western Blot Analysis

The total protein was isolated by the RIPA lysis buffer (Solarbio) according to the instruction procedures, which was quantified by bicinchoninic acid (BCA) detection kit (Shanghai UCHEM Inc., China). Next, the proteins with suitable concentration were separated by SDS-PAGE gels and then transferred to PVDF membranes (Zhejiang Lianshuo Biotechnology Co., Ltd., China). After blocking with 5% blocking solution, the membranes were incubated with anti-E-cadherin (EP700Y, 1 : 10000, Abcam), anti-N-cadherin (ab245117, 1 : 1000, Abcam), anti-Vimentin (ab20346, 1 : 75, Abcam), and anti-TTYH3 (ab80061, 1 : 1000, Abcam) overnight at 4°C, followed by secondary antibodies for 50 min. Finally, protein expression was detected by ECL chemiluminescence and then analyzed via Gel image processing system.

### 2.14. Luciferase Reporter Assay

Briefly, LINC01574-WT and TTYH3-WT were synthesized by cloning the LINC01574 sequence and 3′-UTR of TTYH3 with miR-6745-binding site into pmirGLO reporter vectors (Promega, USA). LINC01574-MUT and TTYH3-MUT were designed using point mutations of miR-6745-binding sites. For transfection, BC cells were cotransfected with the above vectors and miR-6745 mimics or NC mimics. Dual-Luciferase Reporter System (Promega, USA) was used to test relative luciferase activities.

### 2.15. Statistical Analysis

All the data were analyzed by Statistical Package for Social Sciences 21.0 (SPSS, USA). Kaplan-Meier survival curves were employed to analyze the survival of screened lncRNAs in BC patients. Univariate and multivariate Cox analyses were used to screen the survival-related lncRNAs. Receiver operating characteristic (ROC) analysis was performed to estimate the accuracy of LINC01574 in distinguishing BC. One-way ANOVA followed by Tukey's test was employed to evaluate the differences between the groups. *P* < 0.05 was the criterion of statistically significant.

## 3. Results

### 3.1. LINC01574 Expression Was Elevated in BC and Was Positively Related to Adverse Outcomes

**Figure 1:**
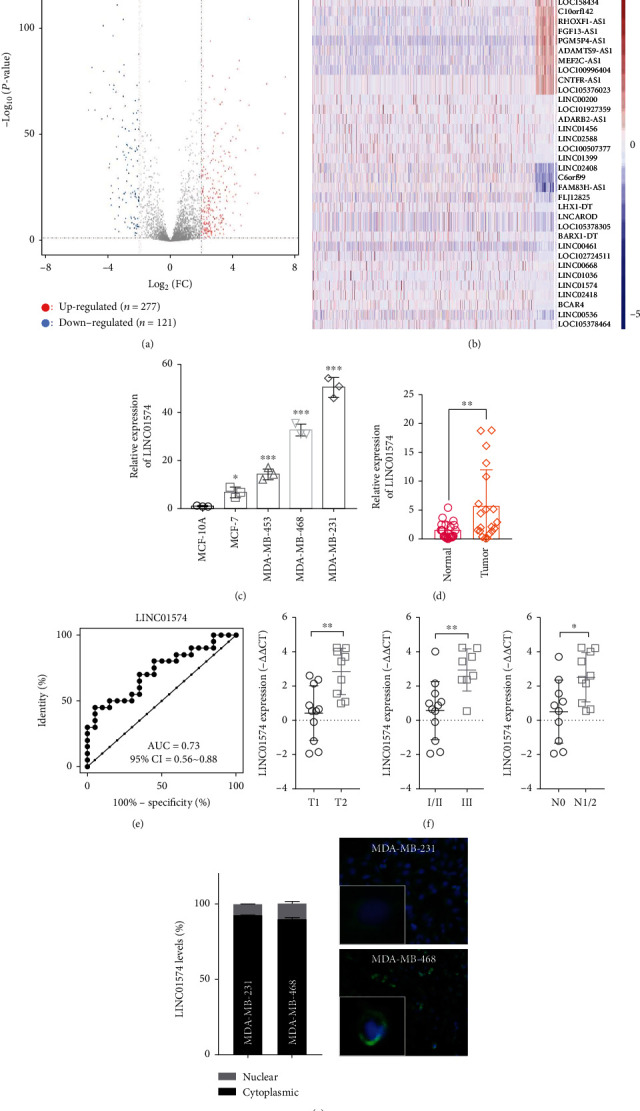
LINC01574 expression was elevated in BC and was positively related to adverse outcomes. (a) Volcano plots showed the differentially expressed lncRNAs between BC tumor and normal tissues. (b) Heat maps showed the OS-related differentially expressed lncRNAs. OS: overall survival; BC: breast cancer. (c) The transcription level of LINC01574 was tested via RT-PCR. (d) The quantitative determination of LINC01574 mRNA level in BC tumor tissues through using qRT-PCR. (e) Receiver operative characteristic of LINC01574. (f) The levels of LINC01574 in different BC tumor sizes, histological stages, and lymph node metastasis. (g) The distributions of LINC01574 in BC cells were analyzed by subcellular localization analysis. ^∗^*P* < 0.05, ^∗∗^*P* < 0.01, and ^∗∗∗^*P* < 0.001.

To identify key lncRNAs involved in BC process, TCGA-BC cohort data were obtained and analyzed. Based on the criteria of ∣logFC | >2 and *P* < 0.05, a total of 398 lncRNAs were evidently different between BC tumor tissues and normal breast tissues, of which 277 lncRNAs were significantly elevated and 121 lncRNAs were sharply decreased (Figure 1(a)). Survival analysis of 398 lncRNAs revealed that 37 dysregulated lncRNAs were related to prognosis in BC (Figure 1(b)). Further, the univariate and multivariate regression analyses were performed for 37 deregulated lncRNAs and clinical features, and we identified a total of 6 independent prognostic risk factors for patient with BC (Table S2). Among them, LINC01574 had the greatest impact on the poor prognosis of BC patients which was screened for further study. Initially, the LINC01574 expression in BC cell lines and BC tumor tissues was analyzed, and we found that LINC01574 was significantly upregulated in BC cells, especially in MDA-MB-231 and MDA-MB-468 (Figure 1(c)). Concomitantly, LINC01574 was also highly expressed in BC tumor tissue than in paired normal tissues (Figure 1(d)). Meanwhile, the ROC algorithm was performed, and the results suggested that LINC01574 has good specificity and can distinguish normal BC tissue from BC tumor tissue (AUC = 0.72, 95%CI = 0.56 − 0.88, Figure 1(e)). Next, the correlation between LINC01574 levels and clinical characteristics was analyzed; we found that the expression of LINC01574 in T2, III, and NI/2 BC tumor tissues was remarkably higher than that in T1, I/II, and N0 BC tumor tissues, respectively (Figure 1(f)). Additionally, the sublocalization of LINC01574 in cells was determined, and in both the cytoplasm and cytoplasm of BC cells, LINC01574 was found, especially in the cytoplasm (Figure 1(g)). Taken together, LINC01574 was evidently upregulated in BC tissues and cells, and its high expression was significantly associated with poor prognosis of BC patients.

### 3.2. Knockdown of LINC01574 Inhibited BC Cell Growth In Vitro and In Vivo

**Figure 2:**
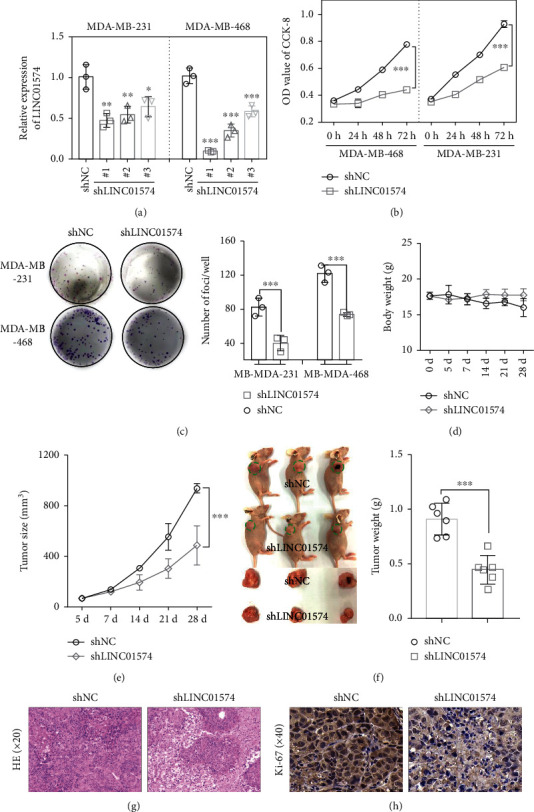
Knockdown of LINC01574 inhibited BC tumor growth *in vitro* and *in vivo*. (a) The mRNA levels of LINC01574 after transfection were tested by qRT-PCR. (b, c) The effects of shLINC01574 on the proliferation of BC cells were examined. (d, e) In the tumorigenesis model, the body weight and tumor size were detected. (f) Tumor volume and weight in the shNC group and shLINC01574 group were assessment. (g, h) H&E and Ki-67 staining in the tumor. ^∗^*P* < 0.05, ^∗∗^*P* < 0.01, and ^∗∗∗^*P* < 0.001.

To investigate the biological function of LINC01574 in BC, we constructed three LINC01574 interference plasmids (shLINC01574#1, shLINC01574#2, and shLINC01574#3) and negative control (shNC) and transfected into the MDA-MB-231 or MDA-MB-468, respectively. In Figure 2(a), all three shLINC01574 significantly inhibited the expression of LINC01574 in comparison to the BC cells transfected with shNC, among which shLINC01574#1 showed the most significant inhibition effect, which was used in the later studies. First, CCK-8 and colony formation were employed to evaluate the effect of LINC01574 knockdown on BC cell proliferation. In CCK-8, we clearly observed that LINC01574 knockdown evidently inhibited the BC cell proliferations in a time-dependent manner (Figure 2(b)). Similarly, colony formation experiments depicted that LINC01574 knockdown remarkably reduced the colony formation (Figure 2(c)). Further, the role of LINC01574 knockdown on tumorigenesis was observed by subcutaneous transplantation in nude mice. During the whole experimental cycle, no statistical difference was presented in body weight between the two groups of nude mice transplanted with shNC-transfected BC cells and shLINC01574-transfected BC cells, but the growth rate of tumor volume in mice transplanted with shLINC01574-transfected BC cells was significantly slowed down, and the tumor volume inhibition rate reached 78% at 28 days (Figures 2(d) and 2(e)). In addition, intuitive morphological observations showed that tumors in nude mice transplanted subcutaneously with shLINC01574-transfected BC cells were significantly smaller than those transplanted with shNC-transfected BC cells. Consistently, the tumor weight of the nude mice transplanted subcutaneously with shLINC01574-transfected BC cells was also remarkably lower than that of mice transplanted with shNC-transfected BC cells (Figure 2(f)). H&E staining and immunohistochemistry showed that LINC01574 knockdown further increased tumor cell death and decreased the Ki-67 protein level in subcutaneous BC tissues (Figures 2(g) and 2(h)). Collectively, the above data indicated that LINC01574 knockdown inhibited BC cell growth in vitro and in vivo.

### 3.3. Knockdown of LINC01574 Inhibited BC Cell Metastasis In Vitro and In Vivo

**Figure 3:**
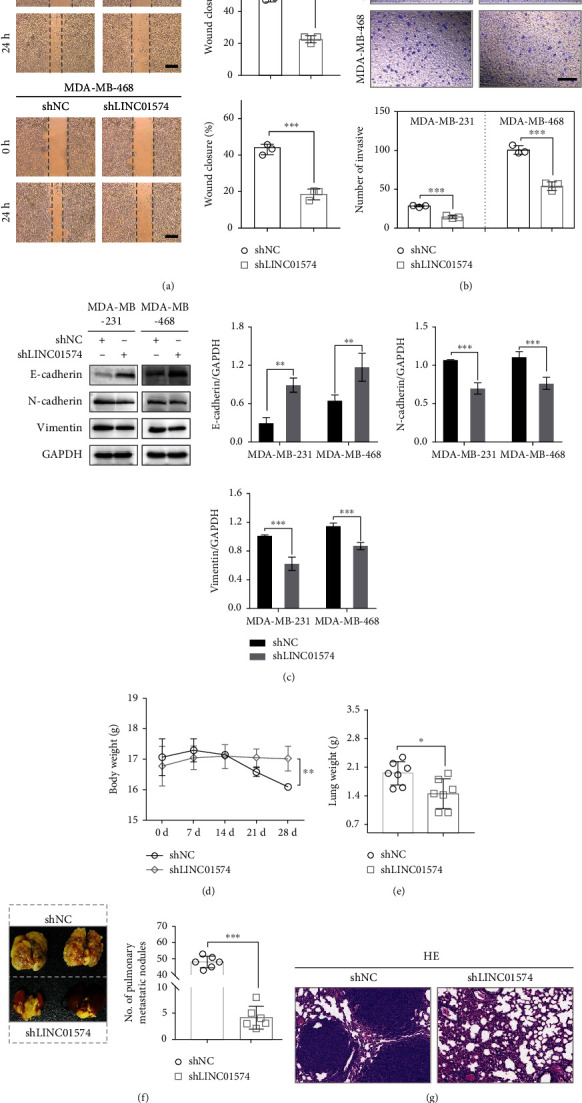
Knockdown of LINC01574 inhibited BC tumor metastasis *in vitro* and *in vivo*. (a, b) After being transfected with shNC or shLINC01574, the migration and invasion capabilities were assessed. (c) EMT-related proteins were detected by western blot. (d, e) In the lung metastasis model, the body weight and tumor weight of nude mice were measured. (f, g) Lungs H&E staining.^∗^*P* < 0.05, ^∗∗^*P* < 0.01, and ^∗∗∗^*P* < 0.001.

Next, the effects of LINC01574 knockdown on the tumor metastasis were assessed in vitro and in vivo. In wound healing assay, BC cells exhibited a wider relative wound healing distance in the shLINC01574 group compared with the shNC group (Figure 3(a)). In transwell invasive assay, we also observed that the invasion number of BC cells transfected shLINC01574 was evidently lower than that of the shNC group (Figure 3(b)). Besides, the protein level of EMT-related genes was detected, and the results depicted that silencing LINC01574 sharply reduced the protein expression of N-cadherin and Vimentin and conversely elevated the protein expression of E-cadherin (Figure 3(c)). Further, a lung metastasis model was used to observe the effect of silencing LINC01574 on lung metastasis of BC cells in vivo. As depicted in Figures 3(d)–3(f), the body weight, lung tissue weight, and lung metastasis number in the shLINC01574 group were remarkably lower than those in the shNC group. H&E staining further confirmed that silencing LINC01574 significantly suppressed lung metastases of BC cells (Figure 3(g)). Collectively, LINC01574 knockdown inhibited BC cell metastasis in vitro and in vivo.

### 3.4. miR-6745 Regulated by LINC01574 Inhibited BC Cell Proliferation, Migration, and Invasion

**Figure 4:**
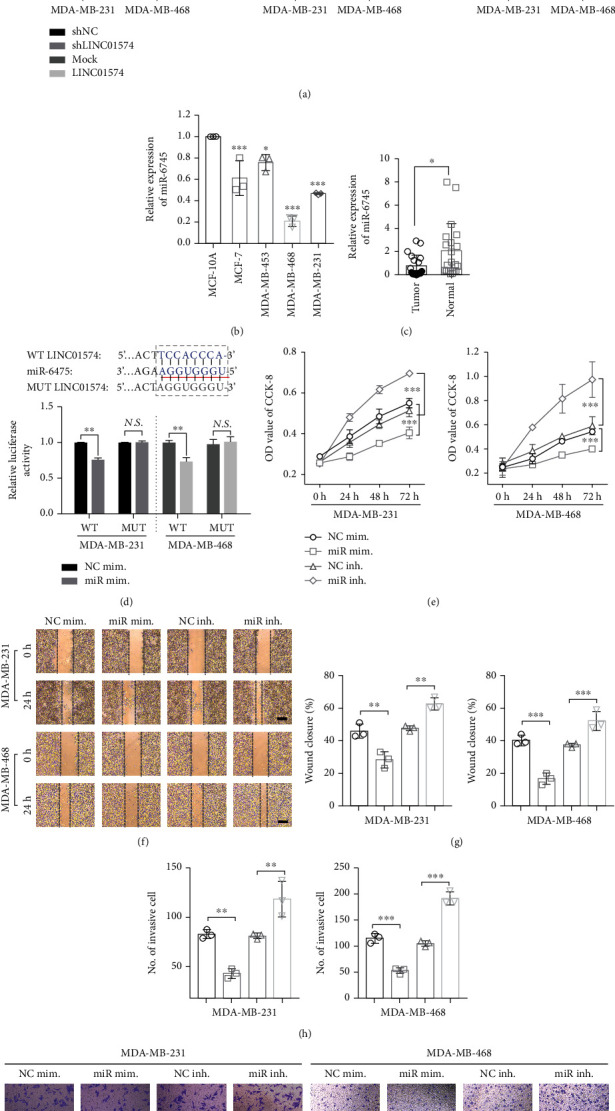
miR-6745 regulated by LINC01574 inhibited BC cell proliferation, migration, and invasion. (a) The mRNA level of miR-6745, miR-633-5p, and miR-4291 was detected by qRT-PCR. (b, c) Relative expression of miR-6745 in BC cell tumor tissues. (d) After WT-LINC01574 or MUT-LINC01574 were cotransfected with miR-6745 mimics, the relative luciferase activity was evaluated. (e–i) After corresponding transfection, the proliferation, migration, and invasion abilities were detected. ^∗^*P* < 0.05, ^∗∗^*P* < 0.01, and ^∗∗∗^*P* < 0.001.

Since LINC01574 was mainly positioned in the cytoplasm, we speculated that LINC01574 may act as a miRNA sponge. To test this hypothesis, the potential targets of LINC01574 were predicted by miRBD, and three miRNAs with scores greater than 70 were screened, including miR-6745, miR-363-5p, and miR-4291. The results of qRT-PCR revealed that overexpression of LINC01574 evidently decreased the expression of miR-6745 in BC cells, and silencing LINC01574 could significantly increase the expression of miR-6745. However, overexpression or silencing of LINC01574 did not affect miR-363-5p and miR-4291 (Figure 4(a)). Further, we found that the miR-6475 was also evidently downregulated in BC tissues and cells (Figures 4(b) and 4(c)). To determine the effect of LINC01574 sponge adsorption on miR-6745 expression, two types of luciferase reporter gene vectors were constructed (LINC01574-MUT and LINC01574-WT). As shown in Figure 4(d), after transfection of miR-6745 mimics, the relative luciferase activity in BC cells cotransfected with miR-6745 mimics and LINC01574-WT was significantly lower than that of BC cells cotransfected with NC mimics and LINC01574-WT remarkably, whereas the relative luciferase activity in BC cells cotransfected with miR-6745 mimics and LINC01574-MUT was not different from that of BC cells cotransfected with NC mimics and LINC01574-MUT.

To further excavate the effect of miR-6745 on the behavior of BC cells, we transfected miR-6745 mimics, miR-6745 inhibitor, and the corresponding NC mimics and NC inhibitor into BC cells, respectively. The CCK-8 evidence revealed that overexpressed miR-6745 evidently restrained the proliferation rate of BC cells, while silencing miR-6745 significantly increased the proliferation rate of BC cells (Figure 4(e)). For the wound healing evidence, the high expression of miR-6745 remarkably reduced the wound closure rate of BC cells, while the silencing of miR-6745 conversely increased the migration capacity (Figures 4(f) and 4(g)). Consistently, transwell invasive assay data indicated that miR-6745 overexpression sharply reduced the number of invasive BC cells, while silencing miR-6745 significantly elevated the invasive ability of BC cells (Figures 4(h) and 4(i)). In summary, our data proved that miR-6745 was regulated by LINC01574 and may play a role as a tumor suppressor gene in the development and progression of BC.

### 3.5. LINC01574 Affected TTYH3 Expression by Regulating miR-6745

**Figure 5:**
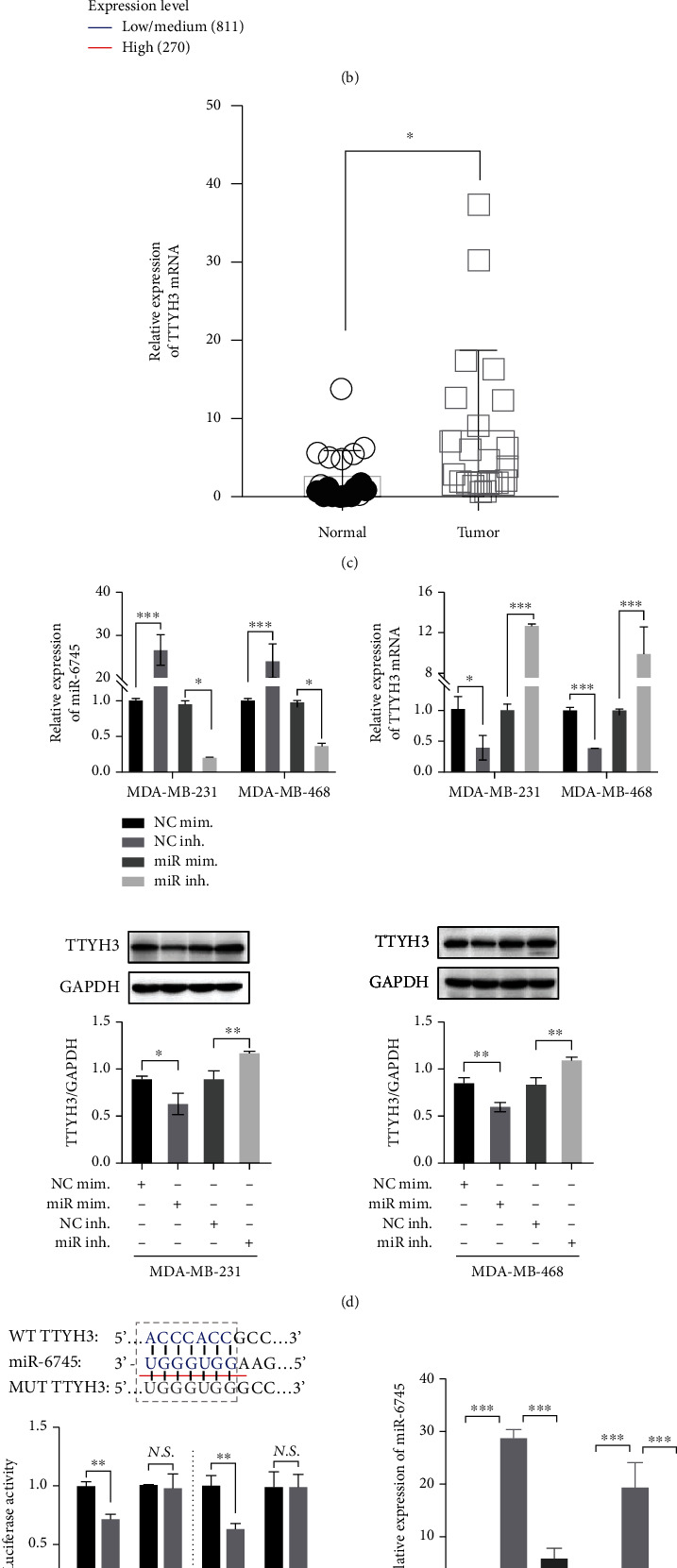
LINC01574 affected TTYH3 expression by regulation of miR-6745. (a) miRWalk, TargetScan7, and miRDB algorithm were used to predicted downstream target mRNAs with potential binding sites for miR-6745. (b) TCGA and CPTAC databases were used to analyze the expression of TTYH3 and survival in BC patients. (c) QRT-PCR quantitatively detected the transcriptional expression of TTYH3 in BC tumor tissues. (d) After corresponding transfection, the mRNA level of miR-6745 and TTYH3 and the protein level of TTYH3 were analyzed. (e) The interaction between miR-6745 and TTYH3 was verified through luciferase reporter assay. (f, g) After corresponding transfection, the mRNA level of miR-6745 and TTYH3 and the protein level of TTYH3 were analyzed. ^∗^*P* < 0.05, ^∗∗^*P* < 0.01, and ^∗∗∗^*P* < 0.001.

To elaborate the regulatory mechanism of miR-6745 in BC, we performed bioinformatics analysis using the miRWalk/TargetScan7/miRDB algorithm and predicted 10 mRNAs with potential binding sites for miRNAs, including TTYH3 (Figure 5(a)). The analysis of the survival data from TCGA through the UALCAN online website showed that just the TTYH3 mRNA level was related to the prognosis of BC patients; that is, the increased expression of TTYH3 in BC tissues predicted poor prognosis of patients with BC. Besides, TCGA data and CPTAC data demonstrated high expression of TTYH3 in BC tissues at both transcriptional and translational levels (Figure 5(b)). Meanwhile, qRT-PCR detection showed significant upregulation of TTYH3 in BC tumor tissues (Figure 5(c)). Thus, we speculated that TTYH3 was the direct target miR-6745. To verify the above speculation, the expression of miR-6745 and TTYH3 was detected after transfection with the corresponding plasmid. qRT-PCR and western blot evidence revealed that the mRNA level of miR-6475 in BC cells transfected with miR-6745 mimic was remarkably upregulated, while the expression of TTYH3 mRNA and protein was sharply decreased. In BC cells transfected with miR-6745 inhibitor, the expression of miR-6475 and TTYH3 showed an opposite trend (Figure 5(d)). Subsequently, TTYH3-MUT and TTYH3-WT were constructed to analyze the luciferase activity. As shown in Figure 5(e), after cotransfection with TTYH3-WTand miR-6745 mimics, the relative luciferase activity of BC cells was sharply decreased compared with cotransfection with TTYH3-WTand NC mimics. However, when the binding site was mutated, overexpressed miR-6745 did not alter luciferase activity. Taken together, these data indicated that TTYH3 was the target of miR-6745.

We have further confirmed whether LINC01574 regulated TTYH3 expression by sponging miR-6745; rescue experiments were performed in vitro. BC cells were transfected with shLINC01574 alone or cotransfected with miR-6745 inhibitor. As shown in Figures 5(f) and 5(g), silencing LINC01574 resulted in high expression of miR-6745 in BC cells, which in turn significantly inhibited the transcription and translation of TTYH3. However, cotransfection of miR-6745 inhibitor and shLINC01574 in BC cells reversed the mRNA and protein levels of TTYH3by transfection of shLINC01574 alone. Taken together, our data revealed that LINC01574 knockdown inhibited TTHY3 expression via upregulating miR-6745.

### 3.6. LINC01574 Knockdown Inhibited BC Cell Proliferation, Migration, and Invasion by Regulating the miR-6745/TTYH3 Axis

**Figure 6:**
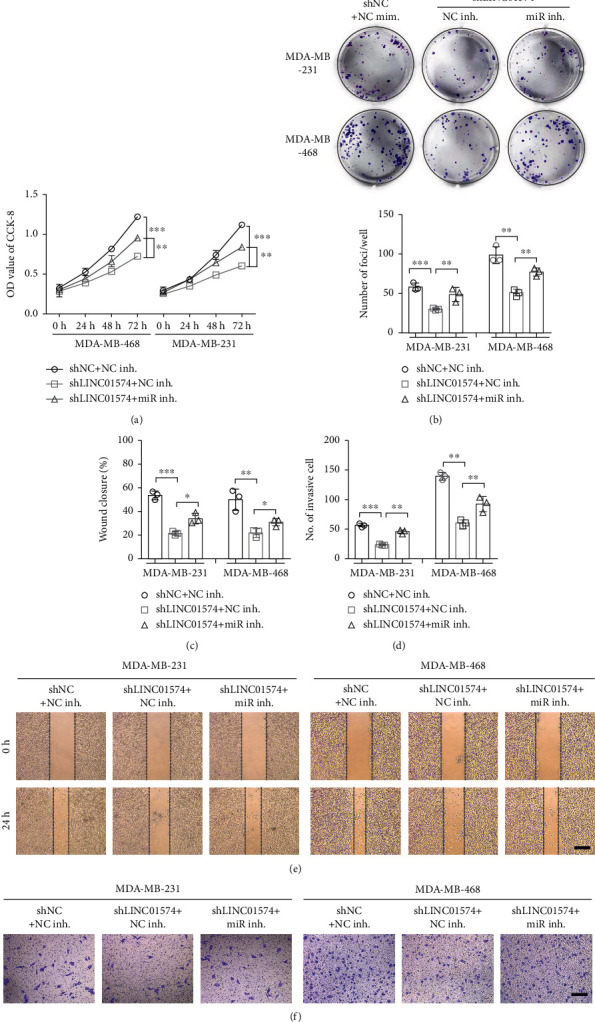
LINC01574 regulated BC cell proliferation, migration, and invasion by targeting miR-6745. (a, b) After corresponding transfection, the OD value and cloning numbers of BC cells were evaluated. (c, e) After corresponding transfection, the wound closure rates of BC cells were tested. (d, f) After corresponding transfection, the number of invasive BC cells was tested.^∗^*P* < 0.05, ^∗∗^*P* < 0.01, and ^∗∗∗^*P* < 0.001.

To determine whether LINC01574 affected BC cell phenotype via regulating the miR-6745/TTYH3 axis, we examined the proliferation, migration, and invasion of BC cells transfected with shLINC01574 alone or cotransfected with miR-6745 inhibitor. The results of CCK-8 and colony formation assay showed that LINC01574 knockdown significantly suppressed the proliferation rate and colony formation of BC cells, while the addition of miR-6745 inhibitor partially attenuated the inhibition effect of shLINC01574 on the proliferation rate and colony formation of BC cells (Figures 6(a) and 6(b)). Consistently, the wound healing and transwell invasion data indicated that LINC01574 knockdown markedly inhibited the migration and invasion abilities of MDA-MB-231 and MDA-MB-468 cells, while the addition of miR-6745 inhibitor partially attenuated the inhibition of shLINC01574 on migration and invasion of BC cells (Figures 6(c)–6(f)).

**Figure 7:**
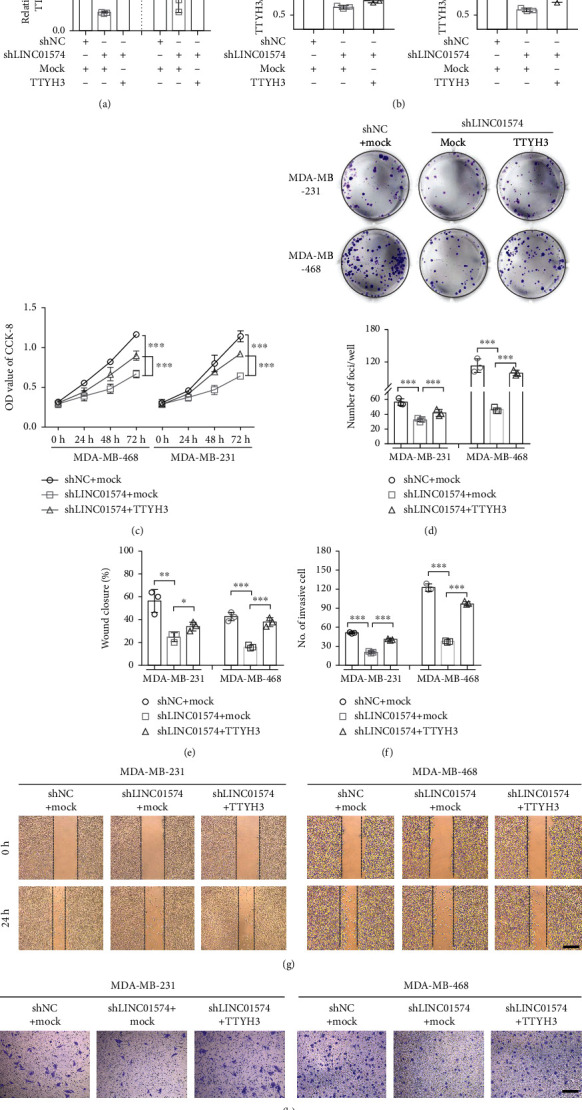
LINC01574 regulated BC cell proliferation, migration, and invasion by targeting TTYH3. (a, b) The transcription and translation levels of TTYH3 were quantitatively detected in BC cells. (c, d) After corresponding transfection, the OD value and cloning numbers of BC cells in different groups (shNC+mock, shLINC01574+mock, and shLINC01574+TTYH3) were evaluated. (e–h) After corresponding transfection, the migration and invasion capabilities of BC cells in different groups (shNC+mock, shLINC01574+mock, and shLINC01574+TTYH3) were evaluated. ^∗^*P* < 0.05, ^∗∗^*P* < 0.01, and ^∗∗∗^*P* < 0.001.

Next, BC cells were transfected with shLINC01574 alone or cotransfected with TTYH3 overexpression vector (TTYH3), and the expression quantification and cell function analysis were also performed. In Figures 7(a) and 7(b), we can clearly observe that the both mRNA and protein levels of TTYH3 in the shLINC01574 group were evidently reduced. Interestingly, the mRNA and protein levels of TTYH3 in the shLINC01574+TTYH3 group were sharply higher than those in the shLINC01574 group. The results of CCK-8 and colony formation assay suggested that LINC01574 knockdown sharply restrained the proliferation rate and colony formation of BC cells, while overexpression of TTYH3 could partially attenuate the inhibitory function of shLINC01574 on the proliferative and colony formation of BC cells (Figures 7(c) and 7(d)). Similarly, the wound healing and transwell invasion data showed that LINC01574 knockdown also remarkably reduced the migration and invasion ability of BC cells, and the inhibitory effect was weakened by TTYH3 overexpression (Figures 7(e)–7(h)). Collectively, these evidences suggested that LINC01574 knockdown inhibited proliferation, migration, and invasion of BC cell by regulating the miR-6745/TTYH3 axis.

## 4. Discussion

It is well known that although lncRNAs lack the ability to encode proteins, they can also regulate gene expression and participate in disease progression in various ways [13]. Recently, many researches have reported the emerging characters of lncRNAs in BC, and they have been shown to be novel gene regulators and prognostic markers of BC. For example, lncRNA-CDC6 was shown to have elevated expression in BC samples and was positively correlated with its malignant stage and poor prognosis [28]. Likewise, LINC01574 was observed to be significantly upregulated in BC, resulting in lower survival rates in our study. Besides, silencing of lncRNA RHPN1-AS1 was proved to decelerate BC deterioration by reducing the proliferative activity and metastatic capacity of BC cells. Notably, shlncRNA RHPN1-AS1 treatment also correspondingly decreased the tumor growth and metastasis in animal models [29]. Consistently, our data also revealed that shLINC01574 treatment can delay BC malignant progression in vivo and in vitro. In most cases, invasion and metastasis of BC, which involves EMT, are the fatal cause of death in patients with BC [30]. Wang et al. indicated that lncRNA SNHG6 accelerated BC cell metastasis by decreasing the protein expression of N-cadherin and Snail and increasing the E-cadherin protein expression [31]. As expected, LINC01574 knockdown also inhibited EMT process by downregulating N-cadherin and Vimentin protein levels and upregulating N-cadherin protein level. Collectively, the above evidences indicated that LINC01574 contributed to the malignant phenotype of BC.

The ceRNA networks reveal the new dimensions of key molecular roles of lncRNAs. Existing data suggested that lncRNAs acted as ceRNAs and counteracted the repressive effects of miRNAs on downstream target gene expression and biological function [32, 33]. For example, lncRNA MEG3 can affect the proliferative and apoptotic phenotype of BC by targeting miR-141-3p/RBMS3 [34]. In this study, we predicted miR-6745 to be a downstream target of LINC01574 by miRBD and further confirmed the direct binding between them by quantitative detection as well as dual fluorescein assay. Currently, there are few data related to miR-6745. A previous article testified that miR-6745 was low expressed in gastric cancer and miR-6745 overexpression reduced the growth of gastric cancer cells in vitro and in vivo, suggesting that miR-6745 might be a tumor suppressor gene in gastric cancer [26]. Surprisingly, we found also that miR-6745 was significantly downregulated in BC, and miR-6745 overexpression reduced proliferative activity and metastatic capacity of BC cells in vitro. Taken together, LINC01574 acted as a sponge for miR-6745 to regulate BC progression.

miRNAs degrade mRNAs or block their translation by base pairing with target gene mRNA-directed silencing complexes, and available data showed that more than 50% of RNA molecules were controlled by miRNAs [35, 36]. Thus, exploring the molecular mechanism of miRNAs in tumorigenesis will contribute to the treatment and diagnosis of tumor. Here, TTYH3 was forecasted to be the direct target of miR-6745 by means of bioinformatics and molecular biology. TTYH3 is a member of the calcium-activated chloride channel family and takes part in plentiful biological processes, particularly in cancer progression. For example, in cholangiocarcinoma, high expression of TTYH3 accelerated ETM transformation and promoted malignant behavior of cholangiocarcinoma cells [37]. In ovarian cancer, cervical cancer, and colorectal cancer, elevated expression of TTYH3 also predicted poor prognosis [38–40]. Interestingly, we found that overexpression of TTYH3 also evidently facilitated the malignant phenotype of BC, manifested by enhanced proliferative and metastatic abilities. Furthermore, the rescue experiments confirmed that silencing LINC01574 attenuated BC cell proliferation, migration, and invasion by regulating the miR-6745/TTYH3 axis.

## 5. Conclusion

In a nutshell, LINC01574 was markedly increased in BC, which was identified as an independent prognostic risk factor of patients with BC. Further, silencing LINC01574 evidently inhibited the growth and metastasis of BC cells by in vitro and vivo experiments. Mechanistically, we demonstrated that silencing LINC01574 relieved the sponge adsorption of miR-6745, promoted the targeted binding of miR-6745 to TTYH3, and inhibited its expression, thereby restraining the proliferation, migration, and invasion ability of BC cells.

## Data Availability

The data used to support the findings of this study are included within the article.

## Supplementary Materials

Supplementary Materials Table S1: summary of patient cohort information from TCGA dataset. Table S2: univariate and multivariate analyses in breast cancer patients from TCGA dataset.
