# Supplementary material for: A Novel Identified Long Intergenic Noncoding RNA, LINC01574, Contributes to Breast Cancer Deterioration via the Regulation of miR-6745/TTYH3 Axis
Source: J Immunol Res. 2022 Jul 27;2022:4201283. doi: 10.1155/2022/4201283 (PMC9348968; doi:10.1155/2022/4201283)
Supplement: Supplementary Materials — Table S1: summary of patient cohort information from TCGA dataset. Table S2: univariate and multivariate analyses in breast cancer patients from TCGA dataset. [file 4201283.f1.docx]

**Supplementary Materials**

**Table S1** Summary of patient cohort information from TCGA dataset.

| **Characteristics** | **Case/number** | **Percentage/%** |
| --- | --- | --- |
| Sex |  |  |
| Male | 12 | 1.10 |
| Female | 1079 | 98.90 |
| Age(years) |  |  |
| Range | 26-90 |  |
| Median | 58 |  |
| Pathological stage |  |  |
| Stage I | 181 | 16.59 |
| Stage II | 619 | 56.74 |
| Stage III | 248 | 22.73 |
| Stage IV | 20 | 1.83 |
| Not known | 23 | 2.11 |
| T stage |  |  |
| T1+T2 | 910 | 83.41 |
| T3 + T4 | 178 | 16.32 |
| Tx | 3 | 0.27 |
| Lymph node status |  |  |
| N0 | 514 | 47.11 |
| N1-2 | 481 | 44.09 |
| N3 | 76 | 6.97 |
| NX | 20 | 1.83 |
| Metastatic |  |  |
| M0 | 908 | 83.23 |
| M1 | 22 | 2.02 |
| MX | 161 | 14.76 |

**Table S2** Univariate and multivariate analyses in breast cancer patients from TCGA dataset.

|  | Univariate analysis | | Multivariate analysis | |
| --- | --- | --- | --- | --- |
| Character | P-value | HR (95% CI) | P-value | HR (95% CI) |
| **ajcc_pathologic_m** | **2.70E-10** | **6.4 (3.6-11)** | **0.036** | **2.21(1.05-4.64)** |
| **ajcc_pathologic_stage** | **8.30E-08** | **2.6 (1.8-3.7)** | **0.001** | **2.79(1.82-4.29)** |
| **LOC105378464** | **9.60E-05** | **2 (1.4-2.9)** | **0.04** | **1.53(1.02-2.29)** |
| **LINC01574** | **0.00042** | **1.9 (1.3-2.8)** | **0.0001** | **2(1.35-2.96)** |
| **LOC100996404** | **0.00093** | **0.55 (0.39-0.78)** | **0.001** | **0.52(0.35-0.77)** |
| **ADARB2-AS1** | **0.0018** | **1.8 (1.2-2.5)** | **0.021** | **1.58(1.07-2.33)** |
| **LINC00668** | **0.0045** | **1.7 (1.2-2.4)** | **0.094** | **1.39(0.95-2.05)** |
| **ADAMTS9-AS1** | **0.0057** | **0.61 (0.43-0.87)** | **0.933** | **0.98(0.63-1.53)** |
| **LINC02613** | **0.007** | **0.62 (0.44-0.88)** | **0.352** | **0.81(0.51-1.27)** |
| **LOC100507377** | **0.0071** | **1.6 (1.1-2.3)** | **0.413** | **1.18(0.79-1.75)** |
| **MEF2C-AS1** | **0.012** | **0.64 (0.45-0.91)** | **0.744** | **0.93(0.6-1.43)** |
| **LINC01456** | **0.012** | **1.6 (1.1-2.4)** | **0.819** | **1.05(0.68-1.63)** |
| **LHX1-DT** | **0.013** | **1.5 (1.1-2.2)** | **0.144** | **0.74(0.49-1.11)** |
| **LINC02588** | **0.015** | **1.5 (1.1-2.2)** | **0.658** | **0.92(0.62-1.35)** |
| **LINC00200** | **0.016** | **1.6 (1.1-2.2)** | **0.284** | **1.25(0.83-1.89)** |
| **PGM5P4-AS1** | **0.021** | **0.67 (0.47-0.94)** | **0.069** | **0.69(0.46-1.03)** |
| **LINC00536** | **0.022** | **1.5 (1.1-2.2)** | **0.595** | **1.12(0.74-1.69)** |
| **LINC02418** | **0.023** | **1.5 (1.1-2.1)** | **0.197** | **1.29(0.88-1.88)** |
| **ajcc_pathologic_t** | **0.023** | **1.6 (1.1-2.5)** | **0.84** | **1.05(0.65-1.68)** |
| **FAM83H-AS1** | **0.025** | **1.5 (1-2.1)** | **0.729** | **0.93(0.63-1.39)** |
| **LOC101927359** | **0.026** | **1.5 (1-2.1)** | **0.015** | **1.62(1.1-2.38)** |
| **KCNIP1-OT1** | **0.027** | **0.68 (0.48-0.96)** | **0.089** | **0.71(0.48-1.05)** |
| **BCAR4** | **0.027** | **1.5 (1-2.1)** | **0.033** | **1.54(1.03-2.28)** |
| **FLJ12825** | **0.028** | **1.5 (1-2.1)** | **0.338** | **1.22(0.81-1.83)** |
| **LINC00461** | **0.033** | **1.5 (1-2.1)** | **0.269** | **1.25(0.84-1.86)** |
| **LOC102724511** | **0.035** | **1.6 (1-2.3)** | **0.341** | **1.25(0.79-1.99)** |
| **LOC105374171** | **0.041** | **0.66 (0.44-0.98)** | **0.571** | **0.88(0.57-1.37)** |
| **LINC02408** | **0.041** | **1.4 (1-2)** | **0.157** | **1.32(0.9-1.94)** |
| **LINC01399** | **0.044** | **1.4 (1-2)** | **0.997** | **1(0.67-1.5)** |
| **FGF13-AS1** | **0.045** | **0.7 (0.5-0.99)** | **0.266** | **0.79(0.53-1.19)** |
| **BARX1-DT** | **0.045** | **1.5 (1-2.1)** | **0.071** | **1.47(0.97-2.23)** |
| LOC158434 | 0.054 | 0.67 (0.45-1) |  |  |
| LOC105376023 | 0.079 | 0.73 (0.51-1) |  |  |
| CNTFR-AS1 | 0.079 | 0.73 (0.51-1) |  |  |
| LINC01036 | 0.09 | 1.5 (0.94-2.5) |  |  |
| C6orf99 | 0.12 | 1.3 (0.93-1.9) |  |  |
| C10orf142 | 0.13 | 0.77 (0.54-1.1) |  | |
| ajcc_pathologic_n | 0.16 | 1.4 (0.88-2.1) |  |  |
| RHOXF1-AS1 | 0.21 | 0.8 (0.57-1.1) |  |  |
| LOC105378305 | 0.24 | 1.2 (0.87-1.7) |  |  |
| LNCAROD | 0.24 | 1.2 (0.87-1.7) |  |  |
